# Supplementary figures and images for: Seasonal Variation in Chemical Compositions of Essential Oils Extracted from Lavandin Flowers in the Yun-Gui Plateau of China
Source: Molecules. 2021 Sep 17;26(18):5639. doi: 10.3390/molecules26185639 (PMC8465160; doi:10.3390/molecules26185639)

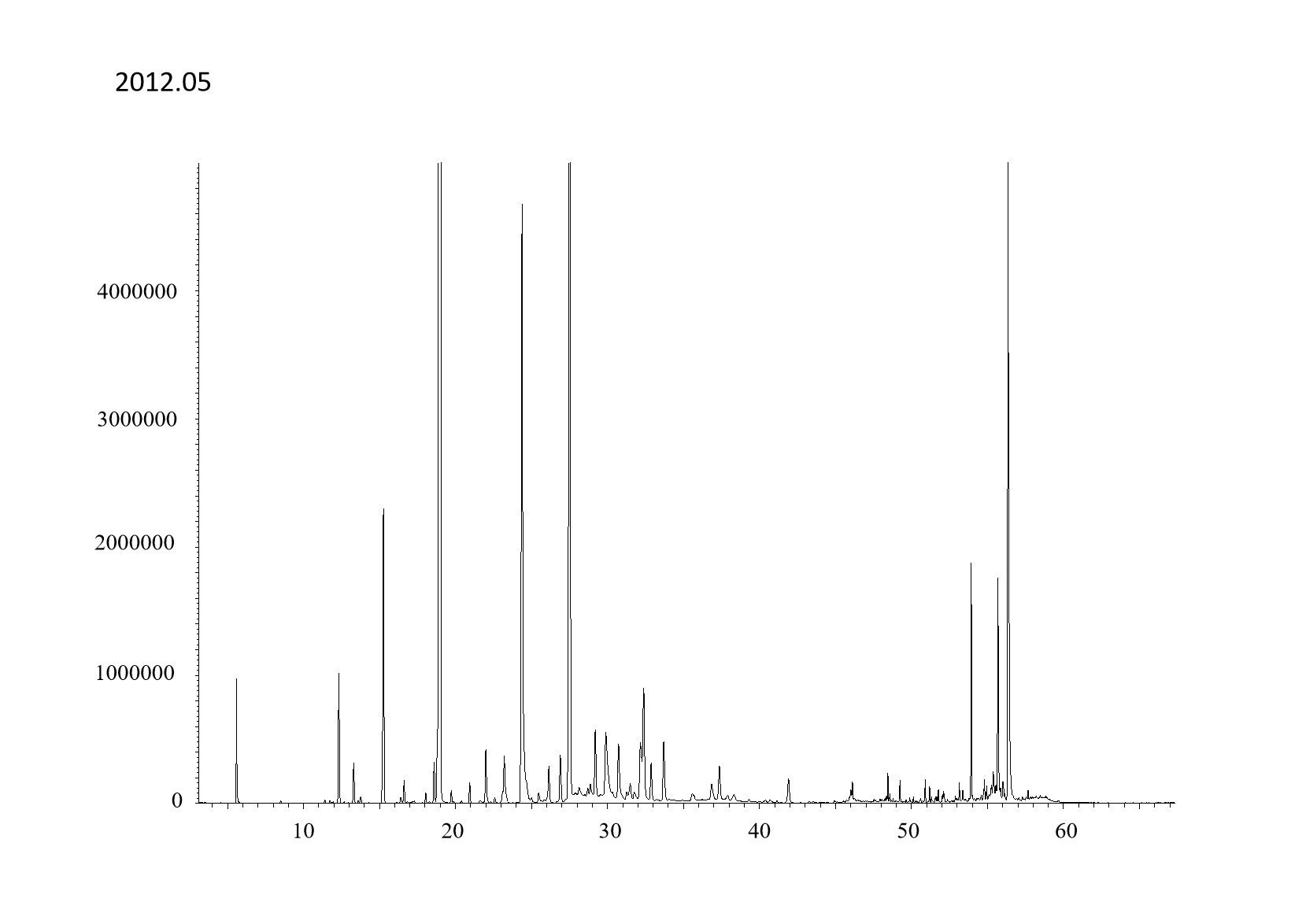

Supplement: Supplementary file 1 [file molecules-26-05639-s001.zip › Supplementary data -The TIC of essential oil from different monthes of L. angustifolia Mill. í┴ L. latifolia Medik/Figure S1 Spring-B TIC.jpg]

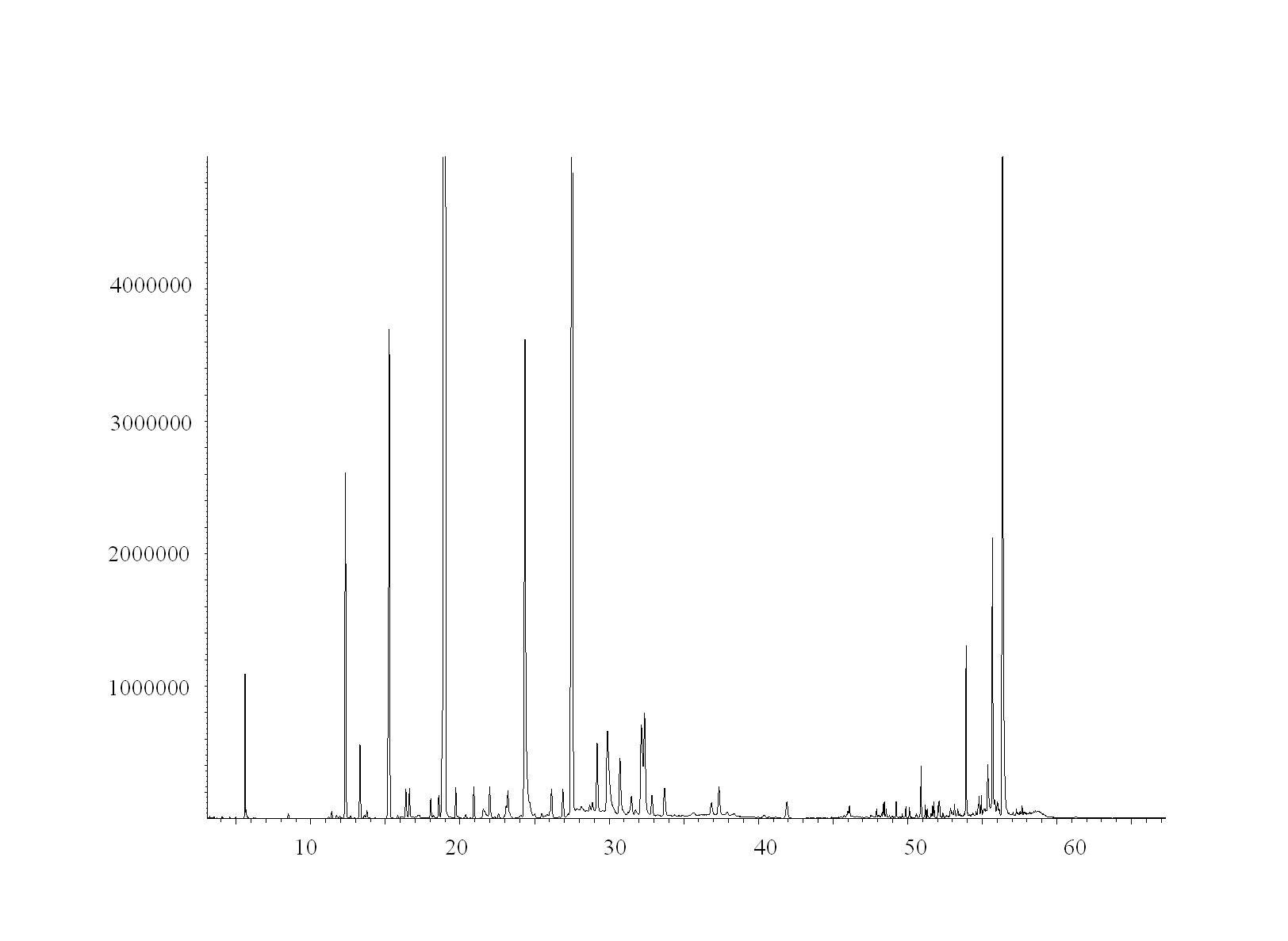

Supplement: Supplementary file 1 [file molecules-26-05639-s001.zip › Supplementary data -The TIC of essential oil from different monthes of L. angustifolia Mill. í┴ L. latifolia Medik/Figure S2 Autumn TIC.jpg]

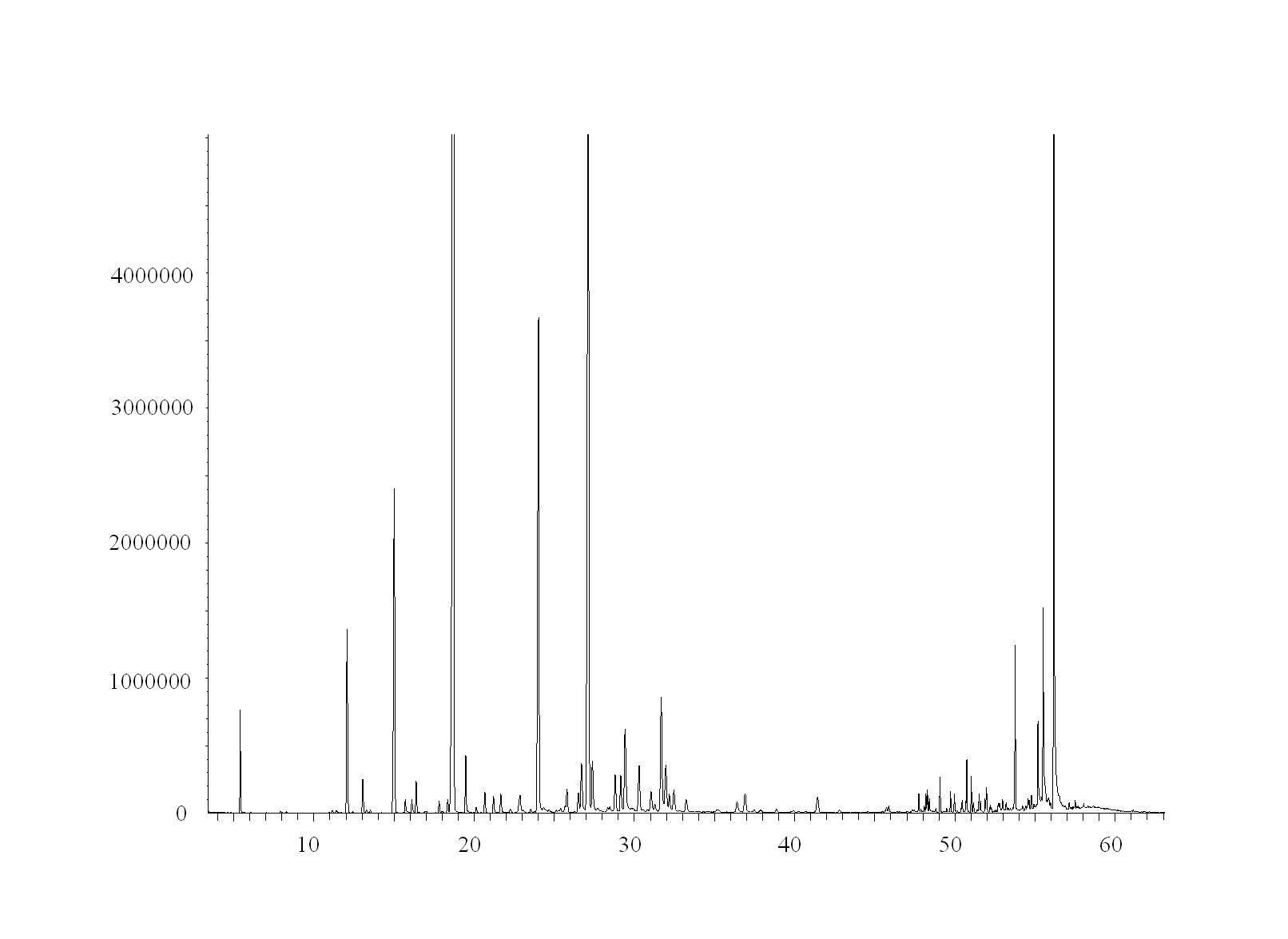

Supplement: Supplementary file 1 [file molecules-26-05639-s001.zip › Supplementary data -The TIC of essential oil from different monthes of L. angustifolia Mill. í┴ L. latifolia Medik/Figure S3 Winter TIC.jpg]

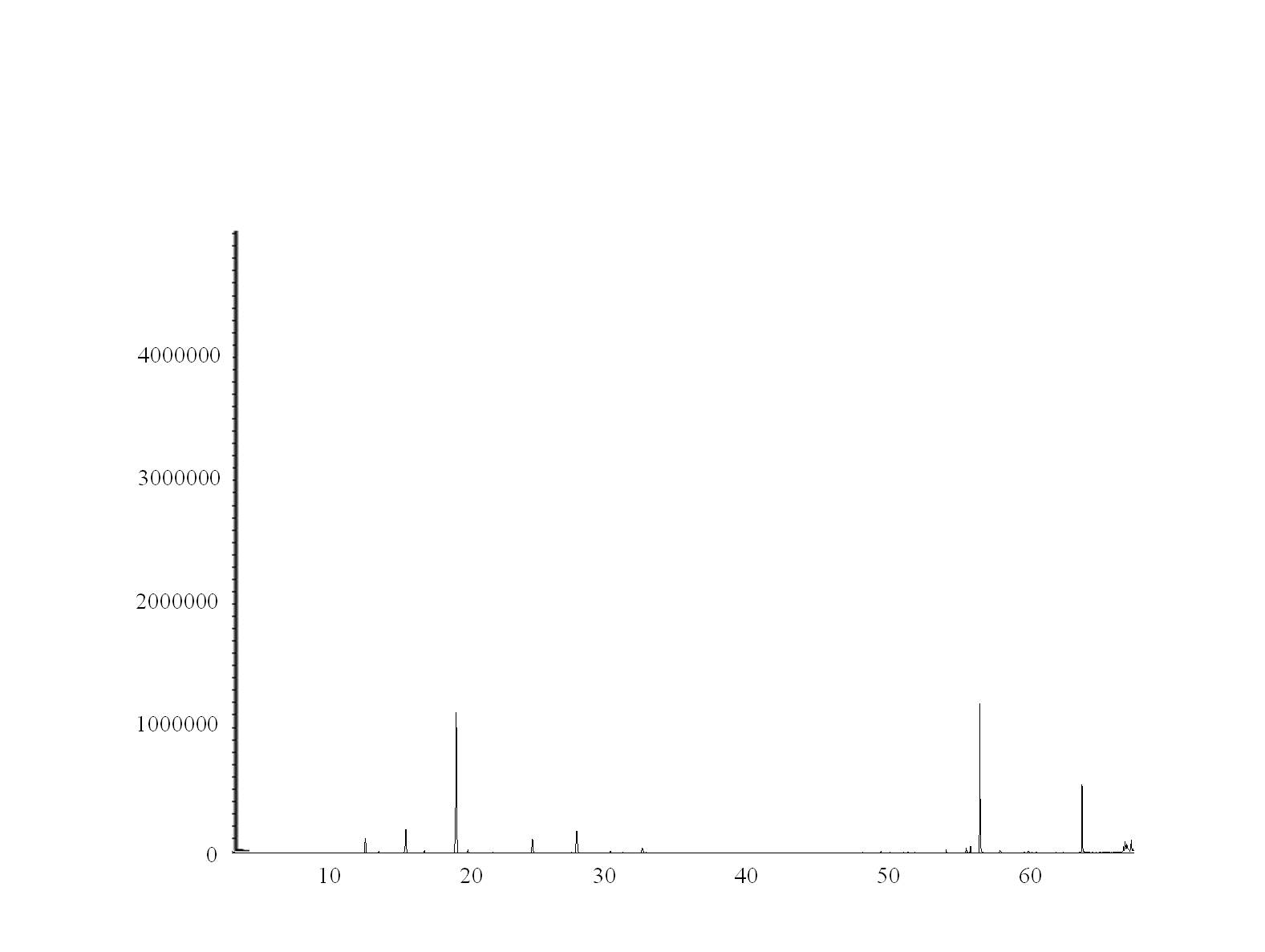

Supplement: Supplementary file 1 [file molecules-26-05639-s001.zip › Supplementary data -The TIC of essential oil from different monthes of L. angustifolia Mill. í┴ L. latifolia Medik/Figure S4 Spring-A TIC.jpg]

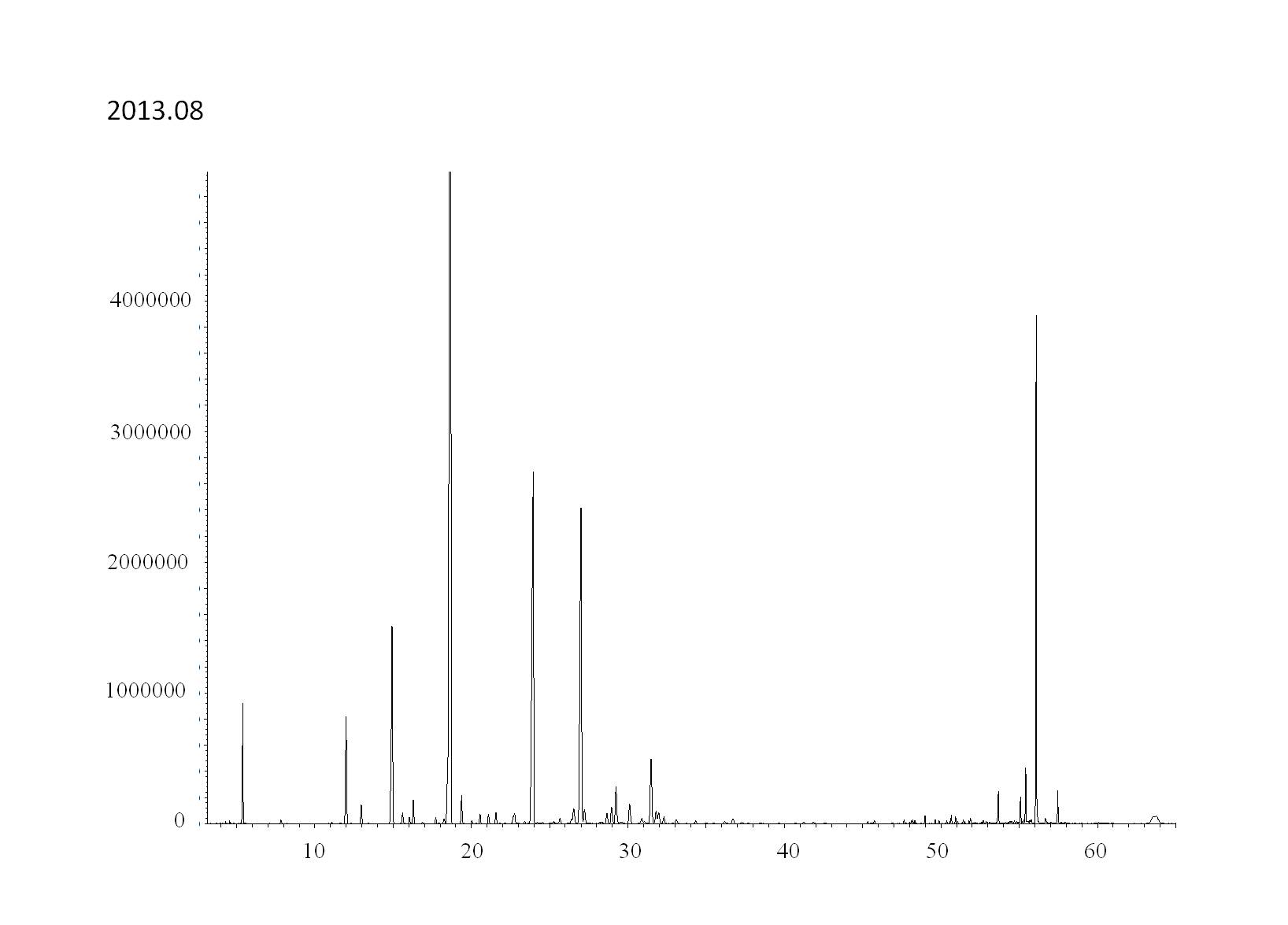

Supplement: Supplementary file 1 [file molecules-26-05639-s001.zip › Supplementary data -The TIC of essential oil from different monthes of L. angustifolia Mill. í┴ L. latifolia Medik/Figure S5 Summer TIC.jpg]

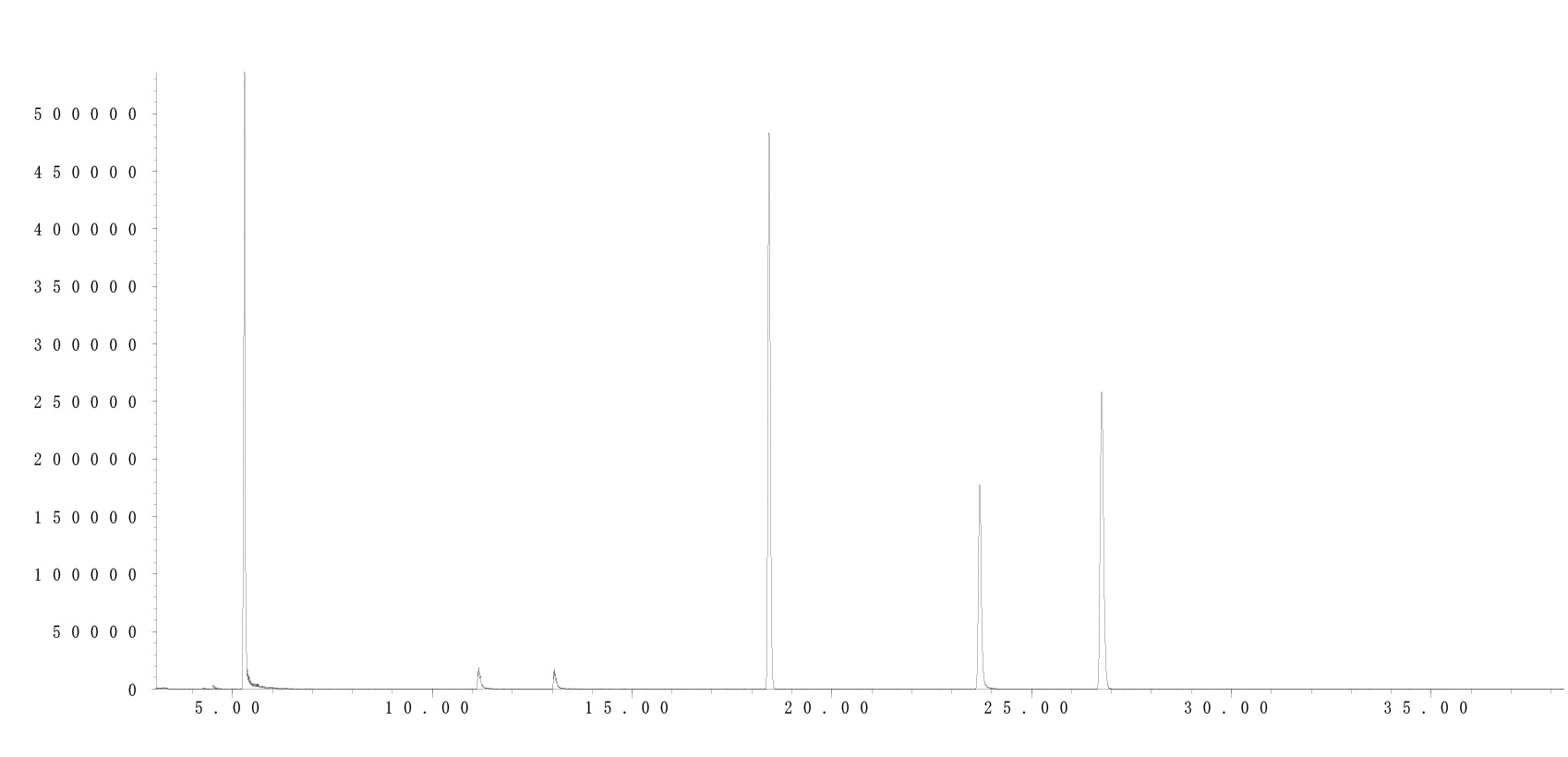

Supplement: Supplementary file 1 [file molecules-26-05639-s001.zip › Supplementary data -The TIC of essential oil from different monthes of L. angustifolia Mill. í┴ L. latifolia Medik/Main compounds Standards TIC/C8-C20.jpg]

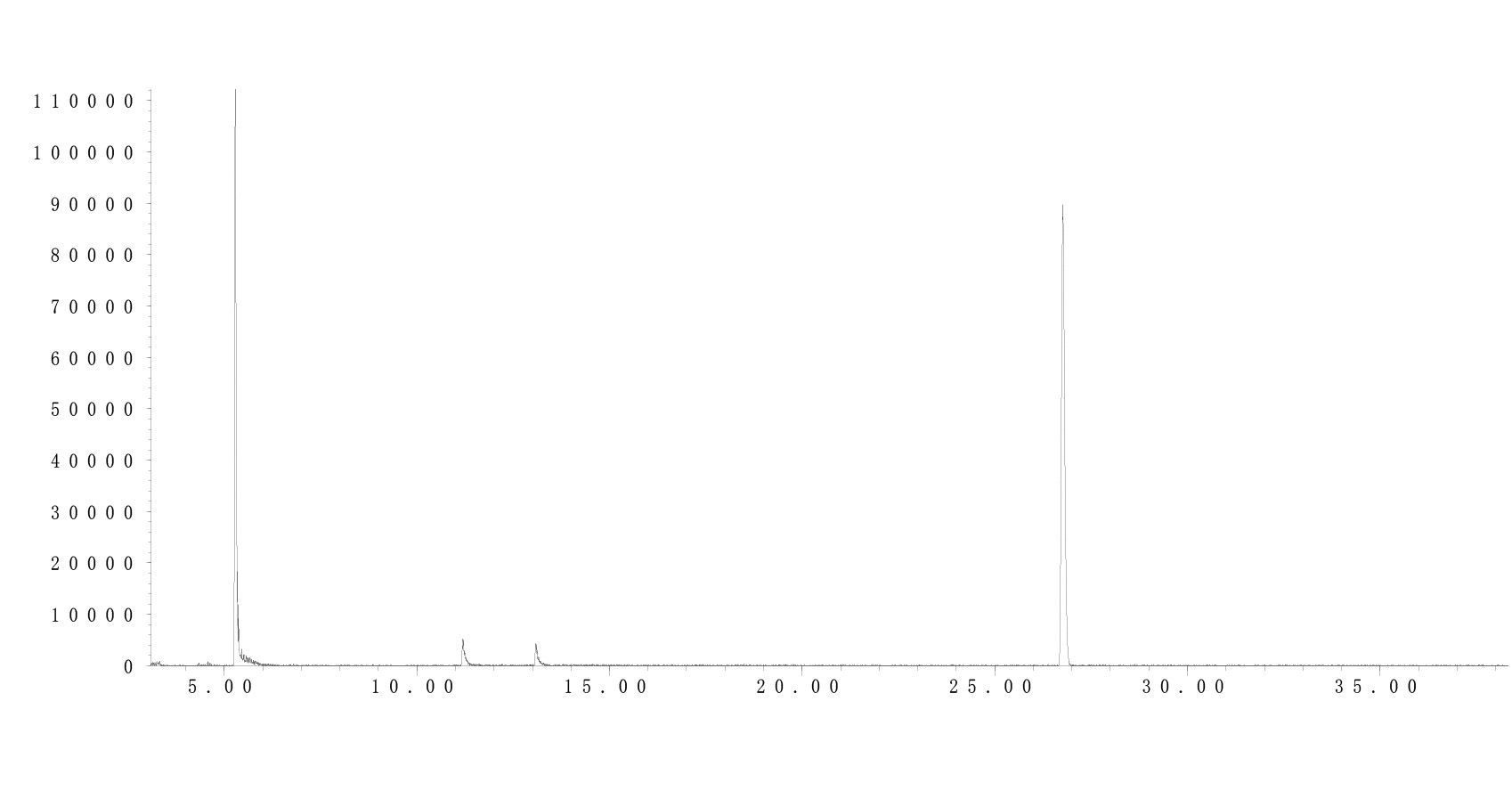

Supplement: Supplementary file 1 [file molecules-26-05639-s001.zip › Supplementary data -The TIC of essential oil from different monthes of L. angustifolia Mill. í┴ L. latifolia Medik/Main compounds Standards TIC/Camphor.jpg]

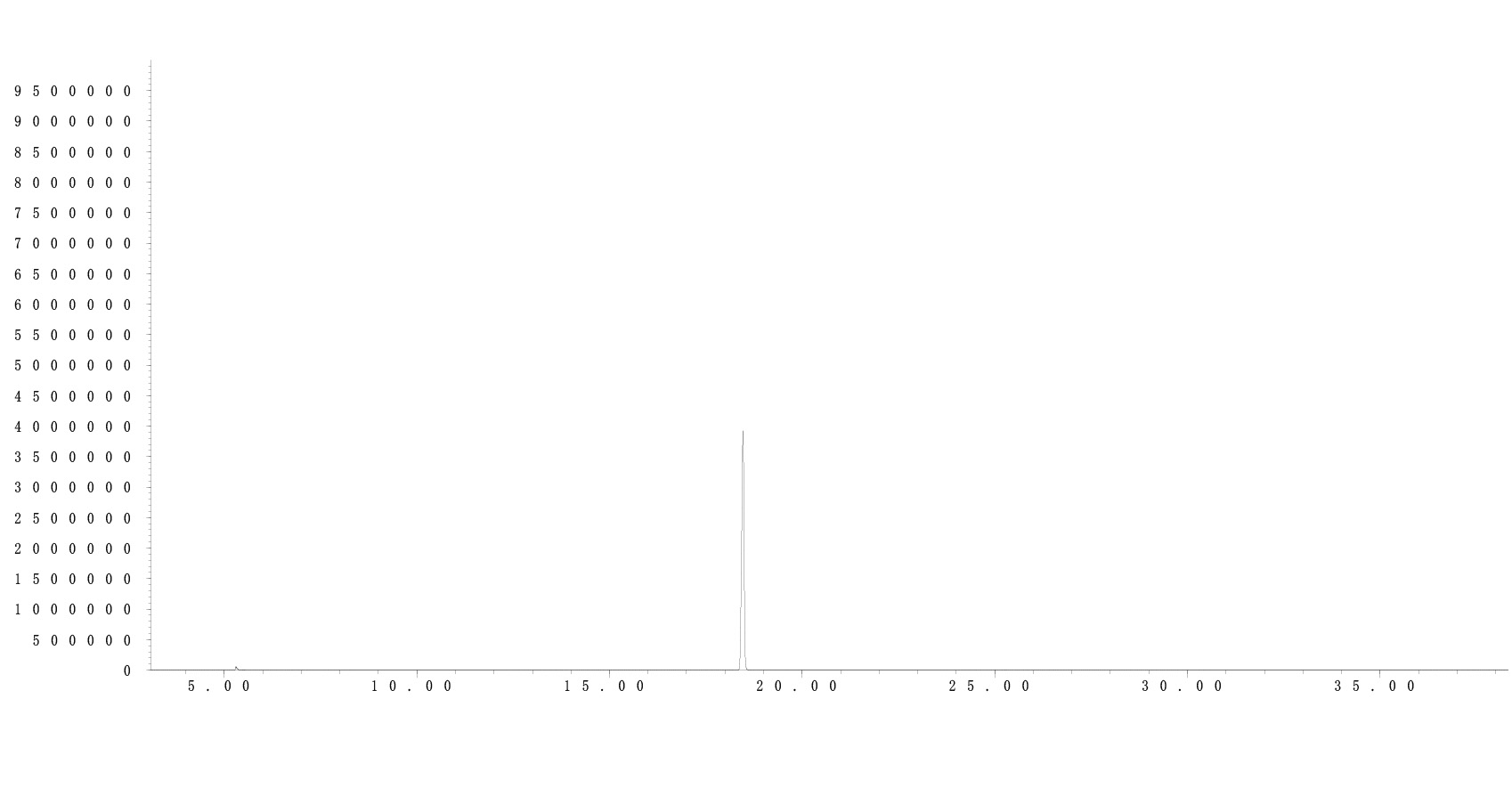

Supplement: Supplementary file 1 [file molecules-26-05639-s001.zip › Supplementary data -The TIC of essential oil from different monthes of L. angustifolia Mill. í┴ L. latifolia Medik/Main compounds Standards TIC/Eucalyptol.jpg]

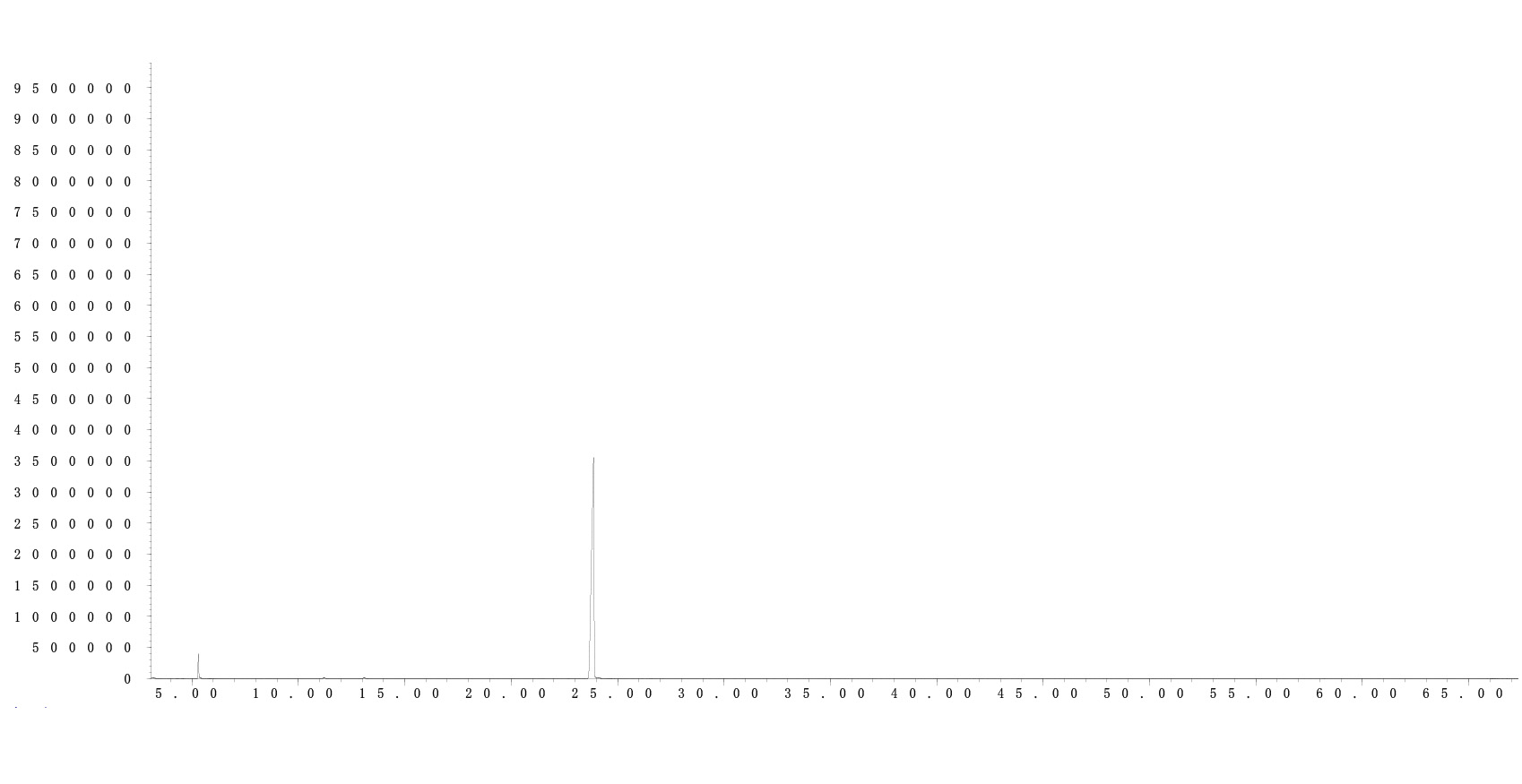

Supplement: Supplementary file 1 [file molecules-26-05639-s001.zip › Supplementary data -The TIC of essential oil from different monthes of L. angustifolia Mill. í┴ L. latifolia Medik/Main compounds Standards TIC/Linalool.jpg]
